# Aortitis and Recurrent Staphylococcus aureus Bacteremia

**DOI:** 10.7759/cureus.57392

**Published:** 2024-04-01

**Authors:** Andreia Coutinho, Inês De Albuquerque Monteiro, Sofia Festa, Sofia Teixeira, Violeta Iglesias

**Affiliations:** 1 Internal Medicine, Centro Hospitalar do Médio Ave, Santo Tirso, PRT

**Keywords:** pancreatic neoplasia, mycotic aneurysm, aortitis due to staphylococcus aureus, recurrent bacteremia, infectious aortitis

## Abstract

Infectious aortitis is a rare entity with high mortality and should be considered in the presence of persistent bacteremia, especially in the absence of endocarditis. We present the clinical case of a woman who developed aortitis due to methicillin-sensitive *Staphylococcus aureus*, complicated with mycotic aneurysm and recurrent bacteremia, even under appropriate treatment. Given the concomitant probable diagnosis of malignant pancreatic neoplasia, the hypothesis of a possible relationship or contribution to bacteremia is raised.

## Introduction

Infectious aortitis and its complications, particularly mycotic aneurysms, have become exceedingly rare with the widespread use of antibiotics [1,2]. The microorganisms most commonly implicated are Staphylococcus, Streptococcus, and Salmonella species. In cases of bacteremia not associated with endocarditis, the aortic artery is a potential focus, favored by the presence of atherosclerotic plaques that facilitate colonization [1,3]. Due to its low incidence and nonspecific symptomatology, diagnosing this condition is challenging. The most common manifestations include fever and pain complaints (chest pain, back pain, or abdominal pain, depending on the level of the infection in the aortic artery). Contrast-enhanced computed tomography (CT) is the diagnostic tool that best allows for identification. Treatment is based on local infection control and management of its complications, along with long-term targeted antibiotic therapy [1,2,4].

## Case presentation

**Figure 1:**
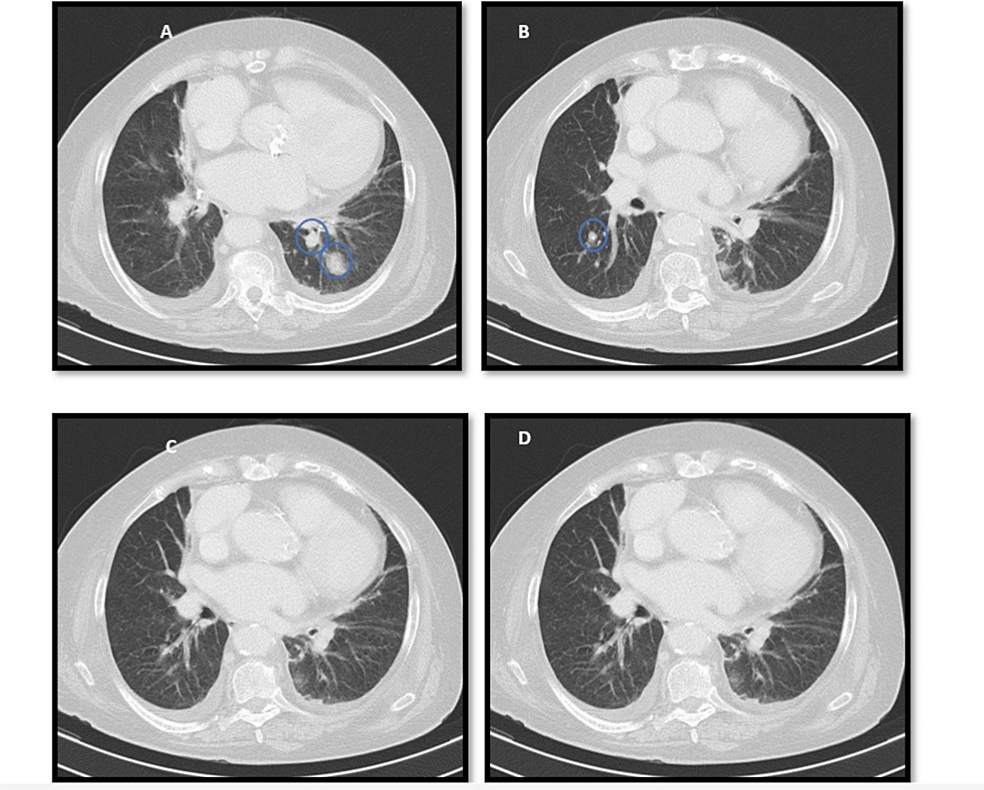
First (A, B) and second (C, D) computed tomography scans. (A, B) Three lesions in the lungs and (C, D) lesions had disappeared.

**Figure 2:**
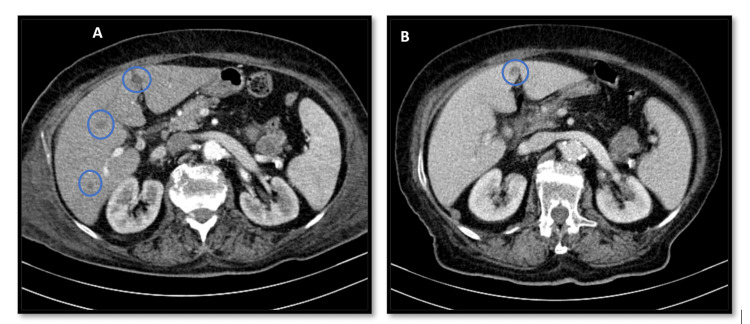
First (A) and second (B) computed tomography scans. (A) Three lesions in the liver and (B) only one is seen.

**Figure 3:**
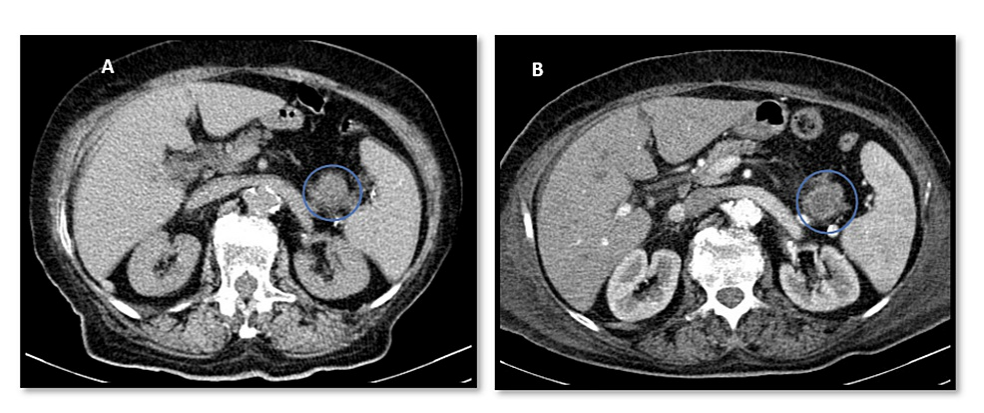
Pancreatic lesion in the first (A) and last (B) computed tomography scans. (B) Lesion is more heterogeneous and has some contrast enhancement.

**Figure 4:**
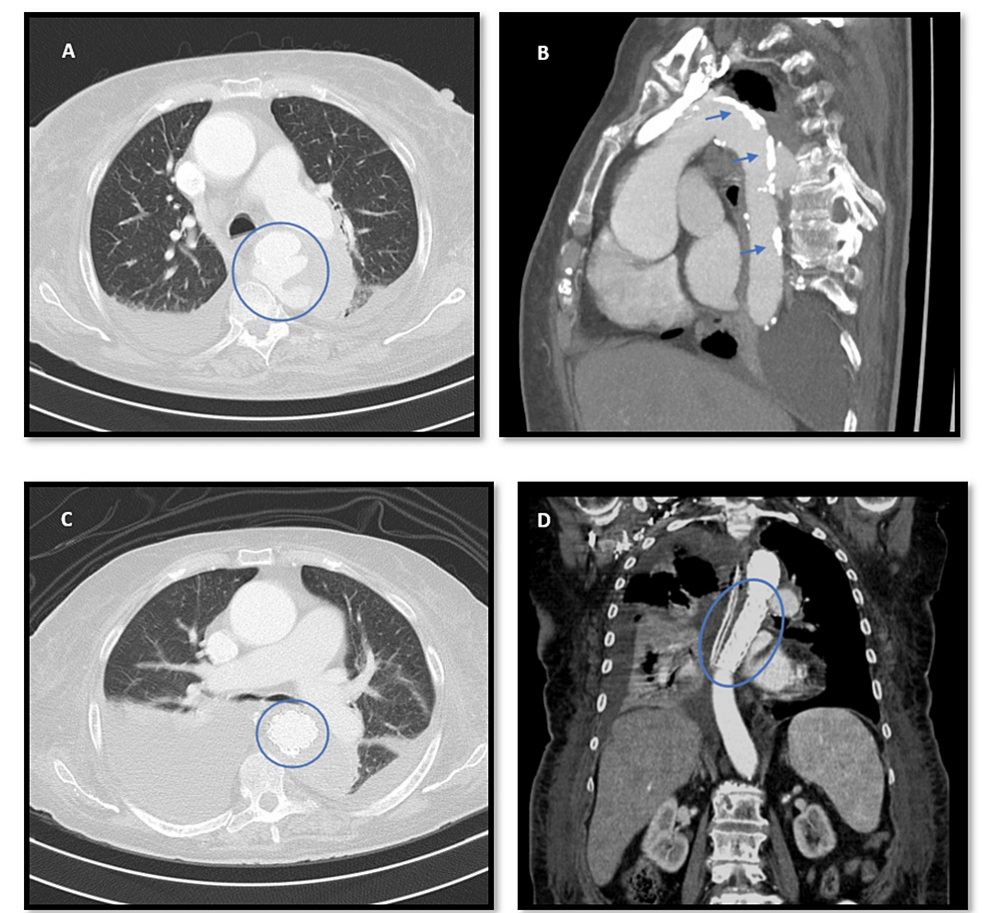
Axial (A) and sagittal (B) computed tomography images of the chest with administration of intravenous contrast agent, where it is documented the Type I Crawford aortic aneurysm. (B) Identifying the atherosclerotic plaque. (C, axial) and (D, coronal) show the repaired aneurysm.

An 81-year-old woman, with heart failure with preserved ejection fraction of valvular/hypertensive etiology, moderate to severe aortic stenosis, arterial hypertension and dyslipidemia, presented to the emergency department with a two-month history of lumbar pain associated with fatigue and anorexia. On examination, her blood pressure was 119/68 mmHg, her heart rate was 140 beats/minute, she had fever (39.2ºC), cutaneous pallor and a systolic aortic murmur (grade II/VI) on cardiac auscultation. The electrocardiogram revealed atrial fibrillation with a rapid ventricular response. Laboratory analysis showed mild normocytic normochromic anaemia (haemoglobin 11.5 g/dL), elevated C-reactive protein (27.78 mg/dL) without leucocytosis, and increased NT-proBNP (7,402 pg/mL). Contrast-enhanced toraco-abdomino-pelvic and spinal computed tomography revealed ascending thoracic aorta ectasia (45 mm) with adjacent tissue densification, three hypotransparent lung lesions (Figures 1A, 1B), three hepatic lesions (Figure 2A), and one in the pancreatic tail (Figure 3A), along with non-recent degenerative changes in the spine. Microbiological studies of urine and respiratory viruses were negative. Due to recurrent fever, blood cultures were obtained, and empirical treatment with ceftriaxone was initiated. The patient was hospitalized for management of fever and elevated inflammatory markers without a clearly identified infectious focus, lumbar pain associated with constitutional symptoms and newly identified ectasia of the aorta and indeterminate-duration atrial fibrillation. Blood cultures identified methicillin-sensitive *Staphylococcus aureus*, and the antibiotic was adjusted to flucloxacillin. Suspicion of infective endocarditis prompted transthoracic and subsequent transoesophageal echocardiograms, but this diagnosis could not be confirmed (there was no evidence of thrombi, vegetations, or abscesses); instead, ascending thoracic aorta dilatation with multiple simple atheroma plaques was emphasized. The patient responded well to instituted antibiotic therapy, with complete resolution of fever and improvement in inflammatory parameters, but an identifiable infectious focus was still lacking. For that reason and for reassessment of the potential septic embolization sites, a repeat computed tomography, 10 days later, revealed a type I Crawford thoracic aortic saccular aneurysm (Figures 4A, 4B) (69x56mm) complicated by intramural ulceration and rupture signs; pulmonary changes had disappeared (Figures 1C, 1D), the liver exhibited only one nodular hypotransparency (Figure 2B) and the pancreatic lesion remained unchanged. Urgent endovascular repair of the aneurysm was successfully performed without complications. Subsequent transoesophageal echocardiogram confirmed no signs of endocarditis, and a new set of blood cultures were sterile. The patient initially had a favourable clinical course, completing six weeks of directed antibiotic therapy. However, she developed progressively worsening and difficult-to-control nausea and vomiting, leading to impaired nutrition, ionic disturbances and progressive deterioration of her general clinical status. Repeated blood cultures remained negative and abdominal CT highlighted the nodular lesion in the pancreatic tail (Figure 3B), now irregular and suspicious of primary neoplastic etiology. An oncology evaluation deemed the patient unfit for further investigation or targeted treatments due to the prolonged hospitalization and patient frailty. The patient's clinical deterioration persisted and ten weeks after admission there was a recurrence of elevated inflammatory markers and positive blood cultures for methicillin-sensitive *S. aureus*, leading to the resumption of antibiotic therapy. Despite directed antibiotic treatment, she continued to have bacteremia four weeks later. At this stage, she was already under palliative care and eventually succumbed 16 weeks after hospital admission.

## Discussion

The patient presented with nonspecific symptoms of fever, lumbar pain, asthenia, and anorexia, which are common symptoms of aortitis. The elevation of inflammatory parameters supported this hypothesis and the findings in contrast-enhanced computed tomography allowed the diagnosis [1,2,4]. This imaging exam is the gold standard for diagnosing aortitis and its complications, where features such as adjacent vessel inflammation, perivascular fluid, and intramural air aneurysm may be present [1,4]. In the first CT, aortic ectasia associated with densification of tissues adjacent to the descending thoracic aorta was observed, reflecting inflammation of the vessel wall. Initially, this finding was not adequately valued, and the hypothesis of endocarditis was pursued. Besides being more common in epidemiological terms, the patient presented one major Duke criterion (positive blood cultures for *S. aureus*) and three minor criteria (fever, degenerative aortic valve disease, and findings consistent with septic embolization). However, consecutive imaging studies (one transthoracic echocardiogram and two transesophageal echocardiograms) failed to reveal changes confirming the diagnosis of infective endocarditis. The mechanisms leading to aortitis include septic embolization, direct bacterial inoculation facilitated by pre-existing changes in the vessel wall (such as atherosclerotic plaque presence), and progression of contiguous infection [1,2,4]. In this case, we understand that the most likely hypothesis was direct inoculation since successive exams failed to demonstrate changes consistent with endocarditis. However, the hypothesis of septic embolization cannot be entirely ruled out. Risk factors for the development of infected aneurysms include trauma, endocarditis, immunosuppression associated with malignant neoplasia, human immunodeficiency virus or diabetes mellitus, and elderly individuals with atherosclerotic disease [1]. This patient had at least the last risk factor mentioned, although we believe that immunosuppression associated with malignant neoplasia could also be present, as will be discussed later. With the progression of the infection in the vessel wall, a mycotic aneurysm forms, a potentially fatal complication that develops rapidly. In this patient, we can clearly observe this evolution, as the second CT, performed 10 days after the first one, already showed the mycotic aneurysm with signs of rupture, even though the patient was under directed antibiotic therapy. Infectious aortitis complicated by mycotic aneurysm has a very high mortality, precisely due to the risk of rupture and massive hemorrhage, especially in the absence of timely surgical/endovascular treatment [3,4]. Thus, survival will depend heavily on a correct and early diagnosis, requiring a high clinical suspicion and well-trained professionals. In this case, the patient received effective antibiotic therapy for 6 weeks and underwent timely endovascular therapy for the mycotic aneurysm, receiving appropriate treatment according to the literature [1,2,4].

Another interesting aspect of this case is the presence of a pancreatic lesion suggestive of neoplasia. In successive imaging studies, the patient showed significant improvement in pulmonary and hepatic lesions under antibiotic therapy, consistent with the hypothesis of septic embolization. However, the pancreatic lesion remained unchanged, and the last CT allowed better characterization, raising the hypothesis of a primary malignant tumor. In this diagnostic hypothesis, we can frame the clinical picture of nausea and vomiting that the patient developed during hospitalization and possibly the constitutional syndrome already presents at admission, as well as the deterioration of her general condition despite adequate infection treatment. There are studies associating different bacterial colonizations or infections with the presence of malignant neoplasms and there is even evidence that patients with *S. aureus* bacteremia have a higher risk of dying from malignant neoplasms in the following year than the general population, namely from pancreatic neoplasms [5,6]. Although, in this case, histological confirmation was not possible, we believe (based on medical history, imaging studies, and clinical evolution) that we are dealing with a highly probable case of pancreatic malignancy. The persistence/recurrence of bacteremia, refractory to directed antibiotic therapy, may have been related to the immunosuppression associated with this neoplastic process, with a consequent higher risk of infectious complications and greater difficulty in controlling the infectious focus, leading to a worse prognosis [3-5]. The patient also had some other mortality risk factors related to aortitis, namely female gender, advanced age, *S. aureus* bacteremia, and aneurysm rupture [7].

## Conclusions

This clinical case represents a rare diagnosis: *S. aureus* bacteremia originating from aortitis and complicated by a mycotic aneurysm. This itself constitutes a challenging diagnosis that should be kept in mind, especially when confirming endocarditis as the infectious focus proves difficult. Additionally, it highlights the presence of persistent/recurrent bacteremia, even under directed antibiotic therapy, which may be associated with the probable diagnosis of pancreatic malignant neoplasia. This association already has some scientific evidence, although further studies are still needed.
